# Bioclipse: an open source workbench for chemo- and bioinformatics

**DOI:** 10.1186/1471-2105-8-59

**Published:** 2007-02-22

**Authors:** Ola Spjuth, Tobias Helmus, Egon L Willighagen, Stefan Kuhn, Martin Eklund, Johannes Wagener, Peter Murray-Rust, Christoph Steinbeck, Jarl ES Wikberg

**Affiliations:** 1 Department of Pharmaceutical Biosciences, Uppsala University, Uppsala, Sweden; 2 Cologne University Bioinformatics Center, Cologne University, Cologne, Germany; 3 Johannes Wagener, Gabelsbergerstr. 58a, 80333 Munich, Germany; 4 Department of Chemistry, Unilever Centre for Molecular Informatics, University of Cambridge, Cambridge, UK

## Abstract

**Background:**

There is a need for software applications that provide users with a complete and extensible toolkit for chemo- and bioinformatics accessible from a single workbench. Commercial packages are expensive and closed source, hence they do not allow end users to modify algorithms and add custom functionality. Existing open source projects are more focused on providing a framework for integrating existing, separately installed bioinformatics packages, rather than providing user-friendly interfaces. No open source chemoinformatics workbench has previously been published, and no sucessful attempts have been made to integrate chemo- and bioinformatics into a single framework.

**Results:**

Bioclipse is an advanced workbench for resources in chemo- and bioinformatics, such as molecules, proteins, sequences, spectra, and scripts. It provides 2D-editing, 3D-visualization, file format conversion, calculation of chemical properties, and much more; all fully integrated into a user-friendly desktop application. Editing supports standard functions such as cut and paste, drag and drop, and undo/redo. Bioclipse is written in Java and based on the Eclipse Rich Client Platform with a state-of-the-art plugin architecture. This gives Bioclipse an advantage over other systems as it can easily be extended with functionality in any desired direction.

**Conclusion:**

Bioclipse is a powerful workbench for bio- and chemoinformatics as well as an advanced integration platform. The rich functionality, intuitive user interface, and powerful plugin architecture make Bioclipse the most advanced and user-friendly open source workbench for chemo- and bioinformatics. Bioclipse is released under Eclipse Public License (EPL), an open source license which sets no constraints on external plugin licensing; it is totally open for both open source plugins as well as commercial ones. Bioclipse is freely available at .

## Background

Chemo- and bioinformatics are important and active research areas with an ever-increasing number of algorithms and software implementations. Numerous applications provide functionality for highly specific tasks, but very few provide a complete one-package solution where all functionality is integrated into a user-friendly workbench. Commercial software like MOE (Chemical Computing Group) and Discovery Suite (Accelrys) contains a lot of features, but are expensive applications, and generally do not allow unlimited extensibility, or have restricted access to source code.

Existing open source projects in bioinformatics that try to solve this problem are usually focused on integrating existing software applications. Jemboss [1] wraps around the EMBOSS [2] collection of open source bioinformatics tools using loose coupling; for example, users can extend functionality by adding shell commands. ISYS [3] is another example of a loosely coupled system that integrates pre-installed applications with a general approach. Gaggle [4] also integrates existing software tools and data sources by wrapping them in code to interchange data between components.

The applications described above are focused on the frameworks for integration rather than providing intuitive interfaces, and are often complex to install, configure, and extend. Few open source projects try to provide an integrated workbench with the possibility for users to add and/or modify functionality without the need to recompile the entire application. Strap [5] is an application for protein alignments that has a workbench with a simple plugin architecture for extension of functionality using the HotSwap [6] mechanism. TOUCAN [7] is a client-server workbench for regulatory sequence analysis using web services, where users can set up and invoke their own algorithmic web services. Taverna [8] is a workbench for dataflow composition and execution where nodes can be web services or components on the local machine which are adapted for use in Taverna. Successful attempts to integrate chemoinformatics and bioinformatics into a single open source framework have not yet been reported.

A workbench in chemo- and bioinformatics should have the required functionality, be easy to install, and have an intuitive and responsive graphical user interface (GUI). Web applications, such as AnaBench [9], are accessed using a browser and run on a remote web server and hence require no local installation. When such applications are run on powerful servers, they perform well on clearly separated and computationally expensive tasks. However, they have restricted client system access and are relatively limited in terms of user interaction, making them unsuitable for integrated applications that require many fast GUI updates. A rich client, in contrast to web-based systems, is a desktop application that takes full advantage of today's computing power in laptops and workstations. It is equipped with a responsive GUI and allows for tight integration with the operating system (e.g. drag and drop, system tray), and usage of local file system and devices (e.g. printers, card readers), but still has the option to invoke and take advantage of remote services and resources (e.g. networked servers, clusters, and databases).

*Bioclipse *is a software project that solves the requirements mentioned above by providing an open source platform for integrating chemo- and bioinformatics into a single framework with an intuitive user interface. Several mature life science frameworks and components are integrated in Bioclipse, and the project actively aims to conform to available standards. The use of an open source license means that anyone can download the source code and make changes, promoting global collaborative development efforts, as well as quick and responsive bug fixing. Bioclipse is part of the Blue Obelisk movement [10], a diverse Internet group that promotes reusable chemistry via open source software development, consistent and complimentary chemoinformatics research, open data, and open standards.

Bioclipse is built on Eclipse [11], a universal tool platform that was originally built as an integrated development environment (IDE), that has evolved over years into a general framework for application development and integration. In Eclipse, all code is split up into plugins, even the core modules. A plugin is a collection of functionality (Java-classes) that can be seamlessly integrated with other plugins, such as algorithms, visualizations, and menu options. This architecture allows for components to be used as building blocks; the minimal set of plugins needed to form a complete application is collectively known as the Rich Client Platform (RCP) [12]. RCP enables software developers to focus on the actual application functionality without concern for standard functionalities, since much of the basic functionality – such as the integration framework and common components – is inherited from Eclipse.

The plugin-architecture of Eclipse is powerful and versatile, and gives developers the ability to add custom functionality to virtually any point in an application. This is a major difference from other plugin architectures, where the user often can only add a pre-compiled class, with limited flexibility, to a pre-determined structure. In Eclipse, it is possible to add views, editors, menus, actions, properties, dialogs, wizards, preferences, help contexts, specify conditions when a certain feature should be available, and even extend the domain object model. That is, the possibilities are endless for extending the program and adapting it to user needs. To define what can be extended, Eclipse utilizes *Extension points*, which exist for almost anything that developers would like to extend, and it is straightforward to create new extension points tailored to user needs.

## Implementation

### Architecture

**Figure 1:**
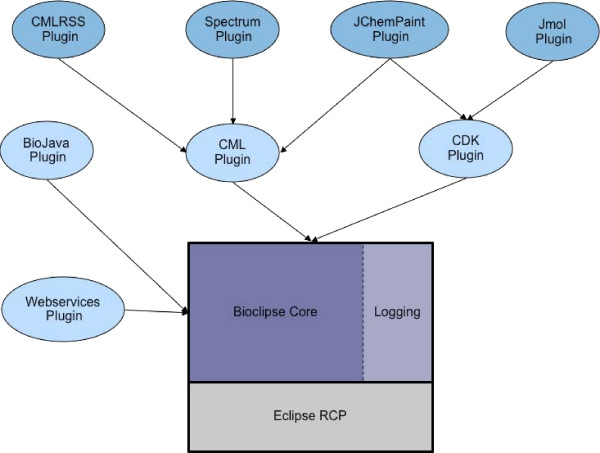
**Plugin dependencies**. Overview of the plugin dependencies for Bioclipse.

Bioclipse is an RCP application extending the Eclipse framework with plugins for chemo- and bioinformatics, and provides a domain-specific platform where the plugins can be integrated (Figure 1). End-users can select desired features from all the Bioclipse plugins, and use them integrated from an intuitive, responsive graphical workbench. For the developer, this is an ideal platform to integrate new components and take advantage of already existing building blocks. Plugins from other Eclipse-based applications could effectively be installed and run within the Bioclipse workbench without modifications, but in order to interact with other components they would require a wrapper for the object model. Bioclipse is developed in Java, a platform-independent programming language which runs on a virtual machine that is freely available for most operating systems (e.g. Windows, Linux, and Mac OS). Several existing application frameworks in bioinformatics have the possibility to add functionality using plugins. However, no previous architecture has the power and flexibility provided by Bioclipse, making it the most advanced integration framework for biosciences available today.

### Object model

**Figure 2:**
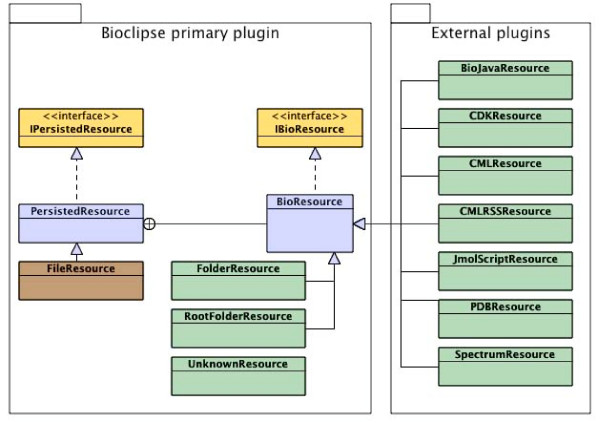
**Object model**. Class diagram of the object model in Bioclipse. Plugins contribute BioResources at runtime using extension points.

The Bioclipse core object model (Figure 2) is defined in the primary plugin, named Bioclipse. This plugin defines a base interface, *IBioResource*, and an implementation, *BioResource*, that implements this interface and provides common properties such as name, path, and size of the current entity. To decouple the resources from their persitence, the interface *IPersistedResource *and the class *PersistedResource *are defined, and the *FileResource *provides a reference implementation for the local file system. This model means that individual plugins need not be concerned with persistence but simply work with IBioResources, unaware if they are located in a database, network, or file.

The primary plugin defines an extension point, *Bioclipse.BioResource*, in order to allow for plugins to extend the core object model. The only extensions to this extension point provided by the primary plugin are the *RootFolderResource, FolderResource*, and *UnknownResource *to mimic folders and files. All specific functionality is contributed by plugins, making Bioclipse a completely modular integration framework (Figure 2). An example implementation of this extension point is the *CDKResource*, contributed by the CDK plugin, that extends the BioResource with functionality for molecular management, supporting various chemical file formats (see below).

### User interface

The Eclipse platform GUI, and hence Bioclipse, is built on SWT (Standard Widget Toolkit). In contrast to Swing/AWT (which provide their own graphical environment), SWT is a native window system; that is, it has the look and feel of the operating system on which the application runs. SWT is designed using the Model View Controller (MVC) software architecture, separating an application's data model, user interface, and control logic into three distinct components so that modifications to one component can be made with minimal impact to others. It is possible to wrap AWT/Swing components in SWT, and this feature is utilized in Bioclipse to integrate Java components built on these toolkits.

In an Eclipse RCP application the user interface is composed of five main graphical units, named *View, Editor, Perspective, Menu *and *Wizard*. A *View *is a window that provides some graphical interface to present a user with information, with the potential to interact with it. An *Editor *is another type of window which is focused on letting the user edit an underlying model and follows the load-save cycle. An example is a simple editor for text files, but could also be a more advanced editor using graphical objects. *Perspectives *are collections of Editors, Views, and Menus that are grouped into a page on screen; one example in Bioclipse is the Chemoinformatics Perspective that displays Views, Editors, and Menu options for working with molecules. *Wizards *are used to guide users through a sequenced set of tasks using different graphical dialogs. The internal placement and size of components within a perspective are not fixed but can be changed at the user's preference and is saved between sessions. Similar to the object model, the primary Bioclipse plugin provides only the most central Views and Editors, while allowing plugins to implement more specialized components.

## Results and discussion

### Features

**Figure 3:**
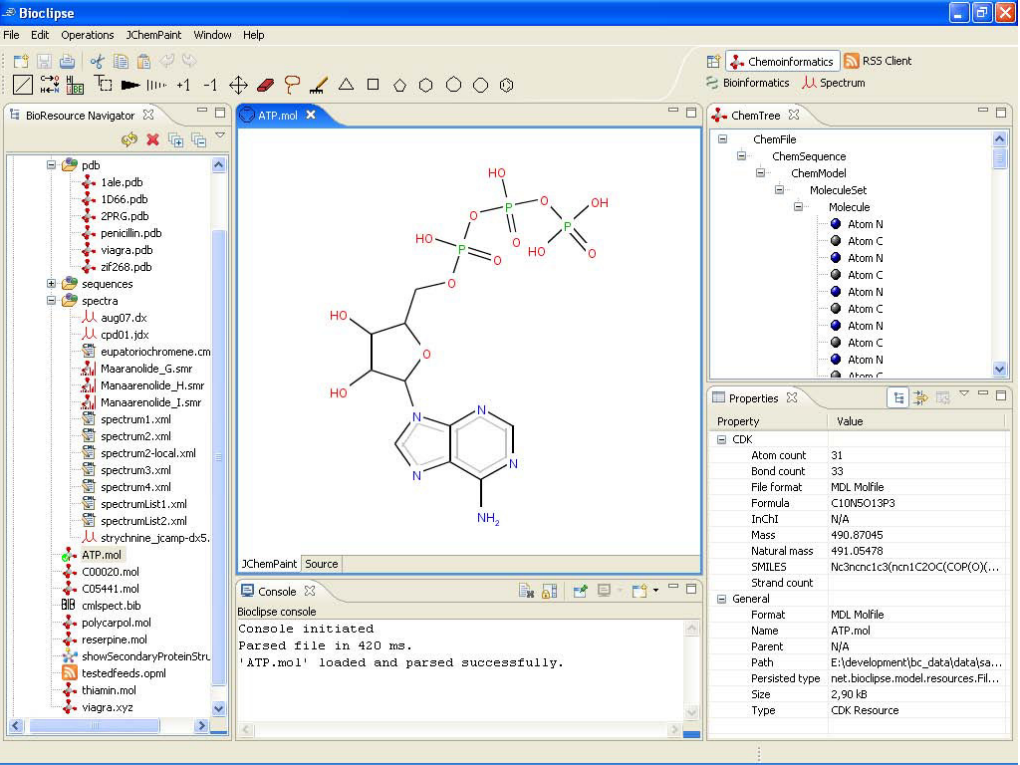
**Screenshot of the Chemoinformatics Perspective**. The Chemoinformatics Perspective showing the BioResource Navigator (on the left), JChemPaint editor with a 2D diagram of ATP (middle), Console echoing messages to the user (bottom), ChemTree view with the atoms and bonds (top right), and the Properties view displaying properties for the selected molecule (bottom right).

The *BioResource Navigator *is the central component in Bioclipse that allows for navigating BioResources in a hierarchical, tree-like View, similar to browsing local folders and files (Figure 3). It contains basic features such as cut and paste, drag and drop, and wizards for new resources, and provides an extension point where plugins can register actions for appearing in the context menu upon right-click. A basic text-editor and an XML-editor with global actions such as undo, redo, cut, and paste are also provided. Bioclipse further contains a *Properties View *for visualization of properties of the currently selected objects in the workbench, a console that echoes messages back to the user, and a job scheduler that allows time-consuming tasks to be run in the background and displayed upon completion (Figure 3). Bioclipse also contains various wizards for the creation of new BioResources, global preferences for customizing the workbench, and a searchable, XML-based help-system that ensures the user manual is readily available – all with extension points so that external plugins can make additions to every part.

#### Chemoinformatics

The *Chemoinformatics Perspective *is a set of Views, Editors, and Menus for molecular management and analysis (Figure 3). Structures are the main data type scientists encounter in chemistry-related fields, and the Chemoinformatics plugins add functionality to Bioclipse that describe chemical structures in various ways.

The *CDK-plugin *integrates the Chemistry Development Kit (CDK) [13,14] library into Bioclipse, and also extends the platform with several graphical components. CDK is a freely available open-source library of Java classes for chemo- and bioinformatics, computational chemistry, and chemometrics. It provides methods for many common tasks in molecular informatics, including 2D and 3D rendering of chemical structures, I/O routines for different chemical file formats, SMILES parsing and generation, QSAR descriptor calculation, atom typing, ring searches, isomorphism checking, and structure diagram generation. The CDK data model for chemical structures is used over the whole platform as an internal data structure for the representation of any kind of molecular data. Bioclipse makes use of the CDK I/O functionality and is capable of writing and reading the same formats for chemical structure information as the CDK itself, which currently are XYZ, MDL molfile, PDB and CML [13,15]. The CDK-plugin adds two views to the bioclipse framework: The ChemTreeView which gives a hierarchical visualization of the CDK data model, and the Structure2DView which displays 2D-Structures.

The *JChemPaint-plugin *provides 2D-editing by wrapping around the JChemPaint editor for 2D molecular structures. JChemPaint is open source, freely available under the LGPL license (GNU Lesser General Public License), completely written in Java, and developed by an international team of developers [16]. The JChemPaint editor is used as the main editor for chemical structures in Bioclipse (Figure 2). It is a Multi-Page Editor which shows two tabs with different views on the same object; The first tab (JChemPaint) displays the structure in 2D and the second (source) shows the molecular data in its original file format. The two tabs are synchronized with each other so that changes in one tab are immediately reflected in the other. The Toolbar and Menu of JChemPaint are directly integrated with the Bioclipse tool- and menu bar. The plugin has the same feature list as the standalone JChemPaint application, including drawing of bonds and atoms, selection of ring templates, flipping and rotating of selected parts of a molecule, undo/redo functionality, and stereo descriptors.

**Figure 4:**
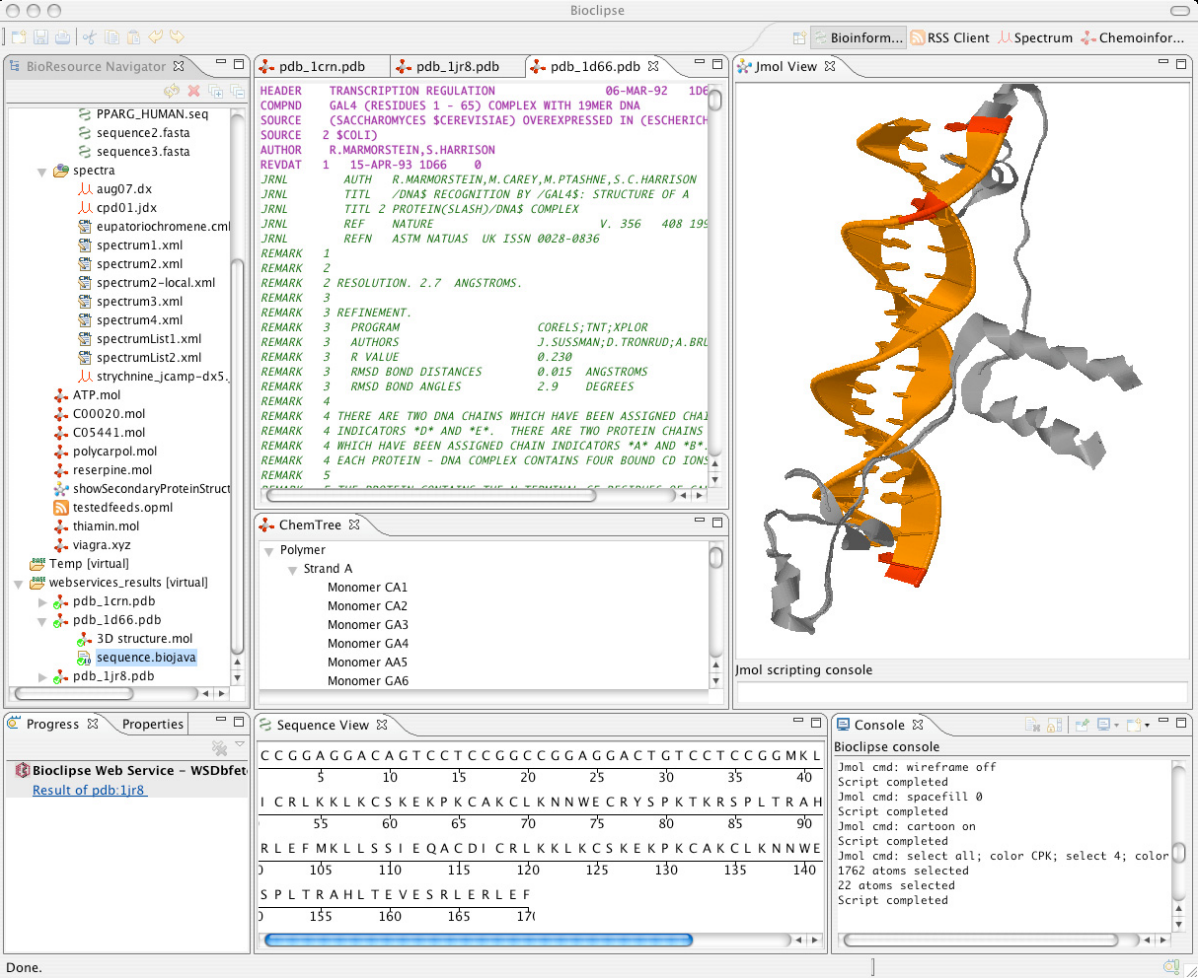
**Screenshot of the Bioinformatics Perspective**. Structure of the protein 1d66 retrieved via an EBI webservice [25]. The BioResource Navigator (on the left) shows a 3D structure and an amino acid sequence object. Selecting residues in the sequence view (bottom) highlights the corresponding structural features in the Jmol view (right).

3D-visualization is provided by the *Jmol-plugin*, wrapping the open source tool Jmol [17] to provide advanced visualization options for molecules and proteins (Figure 4). Jmol includes a scripting language, and Bioclipse offers a console to enter such scripting commands. An Editor for Jmol-scripts is also included that supports editing with code completion and syntax highlighting, as well as the execution of scripts. 

The *CML-plugin *provides access to the Jumbo CML (Chemical Markup Language) library, an open-source Java library for handling and representing CML Documents or -data structures [18]. CML is an XML-implementation for chemical data/information and an extensible basis for chemically aware markup languages. It is structured in a modular way by a core part and several extending components. CML shares all general XML features and advantages, such as data- and not presentation centric, simultaneously human- and machine readable, platform independence, and the ability to represent most general data structures. In Bioclipse, CML is used for the internal representation of spectrum data and for the import and export of structures and spectra to and from the CML file format. Additionally there is an implementation of a CML validation plugin, which checks a given CML file against the CML schema and outputs any detected errors and warnings to the user.

The *CMLRSS-plugin *provides tools for CML-enriched news and blog feeds, supporting the RSS 1.0, RSS 2.0, Atom 0.3 and Atom 1.0 formats [19]. The Bioclipse CMLRSS View automatically extracts CML in the feeds, and resources can directly be visualized and manipulated in Bioclipse. This creates easy access to chemical information published on the web and in databases.

#### Bioinformatics

The *Bioinformatics Perspective *is a collection of Views, Editors, and Menus for loading, parsing, visualizing, editing, converting, and saving sequences/proteins in various formats (Figure 4). Sequence management is provided by BioJava [20], an open-source framework for processing biological data including methods for manipulating biological sequences, file parsers, biological databases, and data analysis routines. A Sequence Viewer can visualize sequences along with SwissProt features. For 3D-visualization of macromolecules, Jmol is also utilized in the Bioinformatics Perspective.

#### Web services

A Web service is a software system designed to support interoperable machine-to-machine interaction over a network. However, the term usually refers to services that use SOAP-formatted [21] XML envelopes and have their interfaces described by the Web Services Description Language (WSDL) [22]. It is becoming increasingly popular for organizations and companies in bioscience to offer such services to provide data access to a public repository, or to invoke remote procedures on a networked computer [23,24].

Bioclipse is equipped with a plugin that allows Web services to be easily integrated into the workbench. The first implementation was the WSDbfetch Web service at the European Bioinformatics Institute, which can return entries from various biological databases [25]. Bioclipse contains a wizard for this service that enables the user to retrieve entries such as PDB-files and sequences in various formats. The retrieved data is then stored in a virtual folder in the BioResource Navigator, parsed and treated as any other BioResource. In the case of an unknown data format, the data is stored as plain text.

#### Spectrum analysis

Compound identification, structure elucidation, and purity control are common tasks in chemistry and biology. Computers can greatly assist in these processes by providing methods for the collection, organization, normalization, and analysis of the data obtained [26]. The *Spectrum-plugin *provides various graphical and non-graphical tools and methods for spectrum visualization, analysis, and manipulation. The plugin contributes the Spectrum perspective, which is mainly formed by three different views with dedicated methods/actions:

• The Spectrum chart views, which use the JFreeChart package [27] for visualization of spectral information (either peak or continuous data). Step-less zoom in/out of the spectrum is possible, as well as setting display properties via the context menu.

• The Metadata View, which displays the stored spectrum meta data in an editable format.

• The PeakTable View, which displays existing peaks in an editable table and gives the user the ability to add, edit, and delete peaks.

The Spectrum plugin comes with routines for importing and exporting data in the CML and JCAMP-DX format, as well as a wizard for the creation of new resources in both formats [18,28]. If continuous data exists for a spectrum, a peak picking action is available for automatic extraction into a peak spectrum. Methods for helping the user with the interpretation of spectra, like calculation of integrals, and comparative views to simplify the direct comparison of different spectra, will be included into the spectrum plugin in a future version. An additional plugin for the assignment of structural to spectral data and vice versa is already in development.

#### Scripting

Bioclipse includes a plugin for creating scripts based on the Mozilla Rhino engine [29]. Rhino is an open-source implementation of JavaScript which is embedded into Java applications to provide scripting to end users. The plugin allows for automation of tasks and creation of new functionality by creating scripts that are able to interact with the GUI, the object model, and features of all installed plugins. Bioclipse is not limited to only one scripting language, and we expect others to be integrated in future releases.

#### Sample data

Bioclipse comes with a plugin for installing sample data including molecules, proteins, sequences, spectra, and scripts in various file formats. Another plugin containing many different organic chemical structures is also included. Installation actions for the available data collections are accessible from the main menu.

## Conclusion

Bioclipse is an advanced open source framework that integrates chemo- and bioinformatics into a single, user-friendly workbench. Equipped with the powerful and versatile plugin architecture of Eclipse, the project has been met with great approval by researchers around the world. Despite its recent introduction, Bioclipse was awarded Third Prize and the audience award at the JAX Innovation Award 2006 [30].

### Bioclipse as workbench

Bioclipse is useful for any user with the need to manage, visualize, and edit chemical and biological files. It is already in use by scientists and teachers in biology, chemistry, and related fields around the world.

### Bioclipse as integration framework

The Bioclipse plugin mechanism facilitates integration of third party applications and libraries. If the software is written in Java, developers can simply implement the desired extension point and link to the library (jar-file). If written in C++, the plugin can use command line invocation or Java Native Interface (JNI), which has been used successfully in Bioclipse. Examples of integrated projects are given in Table 1. Loose connections with external applications using command line invocation are trivial and can be done at runtime using the Bioclipse preferences. They are then immediately available from the context menu in the BioResource Navigator. Bioclipse comes packaged with sample connections to PyMol [31] for 3D-visualization and -rendering, and Strap [5] for sequence alignment.

**Table 1:** Plugins in Bioclipse. A selection of available Bioclipse plugins. For an up-to-date list see the Bioclipse website [34]

bioclipse	Primary plugin
bc_logging	Logging capabilities for other plugins
bc_cdk	Chemoinformatics framework
bc_cml	CML framework
bc_cmlrss	RSS-viewer for CML-enriched feeds
bc_jmol	3D-visualization and scripting
bc_jchempaint	2D molecular editing
bc_biojava	Bioinformatics framework
bc_webservices	Web services framework
bc_spectrum	Spectrum framework
bc_scripting	Scripting framework

### Bioclipse as development environment

Apart from being used for integrating existing projects, Bioclipse is an ideal platform for life science software development and testing. By inheriting all existing functionality of Bioclipse, the developers can focus entirely on the problem at hand, while taking full advantage of the editing and visualization components provided by Bioclipse.

A *Logging-plugin *provides logging functionalities for other plugins. It is based on Log4J [32], a well-designed framework that allows for logging that can be defined and changed at runtime, without modifying the application. Logging is an important feature as it gives developers a detailed context for application failures, and should be provided with most bug reports.

### Bioclipse as deployment platform

Integrating features into Bioclipse is an efficient way of spreading novel algorithms. The simple extension of Bioclipse and the large user base makes any addition readily available for many potential users. Individual contributors thus benefit from existing, as well as forthcoming, additions, which promotes global collaborative development and enables features spanning multiple research fields.

### Future development

The future for Bioclipse holds much potential with many plugins in development, including database persistence, molecular libraries, more Web services, virtual screening, systems biology, phylogenetics, structure elucidation, QSAR, data analysis, R statistical language interoperability, molecular mechanics/dynamics simulations, and 3D-model building. There is also ongoing work for integrating the workflow engine Taverna [8] to run workflows from within the Bioclipse workbench, and use it to present results. Another major feature in development is online updates for plugins from the Bioclipse update server [33]. The current status of the Bioclipse development can be viewed at the Bioclipse website [34] and Bioclipse wiki [35].

The upcoming plugins, powerful plugin architecture, and intuitive interface make Bioclipse the most advanced and user-friendly workbench available today for chemo- and bioinformatics. We encourage software developers in bioscience to consider Bioclipse as a future platform for development and deployment, and welcome new developers, testers, and other contributors to the project.

## Availability and requirements

*Project name*: Bioclipse

*Project home page*: 

*Operating system(s)*: Platform independent

*Programming language*: Java

*Virtual machine*: Sun JVM 1.5.0

*Commercial restrictions*: None

Bioclipse is freely available for download from the project home page.

### License

Bioclipse is released under a custom license EPL + exception, which is the Eclipse Plugin License (EPL) [36] plus the explicit exception that the patent clause of the EPL license does not apply to GPL licensed plugins, addressing the EPL/GPL license conflict [37]. The EPL is a flexible open source license that ensures core plugins will remain open source, but sets no constraints on external plugin licensing. This means that commercial plugins can be developed for Bioclipse, if desired. A list of the licenses for individual plugins and a statement regarding EPL and GPL can be found on the project home page.

## Authors' contributions

OS designed and implemented the core API and extension points and drafted most of the manuscript. CS coordinated the Cologne lab, adapted CDK for Bioclipse, and was involved in manuscript preparation. TH and SK integrated the 2D structure editor JChemPaint and constructed the spectrum plugin. EW worked on the CMLRSS plugin, file format support, and various architectural aspects. ME worked on protein support and API design. JW implemented the webservices- and the scripting plugin. PMR worked on CML support. JESW coordinated the Uppsala lab and was involved in manuscript preparation. All authors performed extensive testing of the application, and approved the final manuscript.
